# Dynamic tensile forces drive collective cell migration through three-dimensional extracellular matrices

**DOI:** 10.1038/srep11458

**Published:** 2015-07-13

**Authors:** Nikolce Gjorevski, Alexandra S. Piotrowski, Victor D. Varner, Celeste M. Nelson

**Affiliations:** 1 Department of Chemical & Biological Engineering, Princeton University, Princeton, NJ 08544, USA; 2 Department of Molecular Biology Princeton University, Princeton, NJ 08544.

## Abstract

Collective cell migration drives tissue remodeling during development, wound repair, and metastatic invasion. The physical mechanisms by which cells move cohesively through dense three-dimensional (3D) extracellular matrix (ECM) remain incompletely understood. Here, we show directly that migration of multicellular cohorts through collagenous matrices occurs via a dynamic pulling mechanism, the nature of which had only been inferred previously in 3D. Tensile forces increase at the invasive front of cohorts, serving a physical, propelling role as well as a regulatory one by conditioning the cells and matrix for further extension. These forces elicit mechanosensitive signaling within the leading edge and align the ECM, creating microtracks conducive to further migration. Moreover, cell movements are highly correlated and in phase with ECM deformations. Migrating cohorts use spatially localized, long-range forces and consequent matrix alignment to navigate through the ECM. These results suggest biophysical forces are critical for 3D collective migration.

Invasive collective migration, in which cells move coordinately through 3D ECM, is a key feature of morphogenesis and wound repair. The tubular structures of branched organs, including the *Drosophila* trachea and vertebrate vasculature, kidney, mammary, and salivary glands^1^,^2^, are generated by large-scale collective cell movements, tightly orchestrated spatially and temporally. These collective movements are also observed during the invasion and metastatic spread of tumor cohorts^3^,^4^,^5^. While cell movement is thought to be initiated and driven by a variety of soluble cues^6^,^7^, collective migration is fundamentally a physical process wherein cells persistently penetrate a dense fibrillar matrix. During the migration of tumor cohorts through collagenous ECM, leader cells propel themselves forward by physically engaging with collagen fibers at the leading edge, and proteolytically processing them at the cell posterior, leaving behind aligned ‘microtracks’ along which follower cells can migrate^8^. A similar mechanism for collective migration has been observed in 3D cocultures of carcinoma cells and fibroblasts, where fibroblasts act as leader cells and create tracks along which cancer cells follow^9^.

The adhesive interactions with the ECM and the indispensable role of integrins^10^ and Rho signaling observed in these and other studies in both two-dimensional (2D) and 3D systems^11^,^12^,^13^,^14^ strongly suggest that collective migration in these cases requires mechanical force. Mechanical forces that arise during collective migration of cellular sheets along flat surfaces and their spatiotemporal variations have been characterized extensively^13^,^14^. However, although they are highly informative about collective behaviors, these models do not fully replicate the mechanical, structural and geometrical features of inherently 3D collective migration processes, including angiogenesis, branching morphogenesis and most cases of cancer invasion. In particular, cellular sheets crawling on surfaces grip the underlying substratum tangentially to propel themselves forward, unconstrained by frontal physical obstacles. In contrast, a 3D matrix provides physical support to an invading cellular collective, but also impedes movement by providing frontal constraint. Furthermore, whereas the mechanically defined materials used in 2D studies enable full quantification of cellular tractions, they do not faithfully mimic the complexity of physiological matrices that respond to these very tractions by altering their structural and mechanical properties^15^,^16^,^17^, likely influencing the migration process. We thus set out to characterize the forces and ECM deformations arising during collective migration through physiological 3D matrices, which had not been fully elucidated previously.

## Results

**Figure 1:**
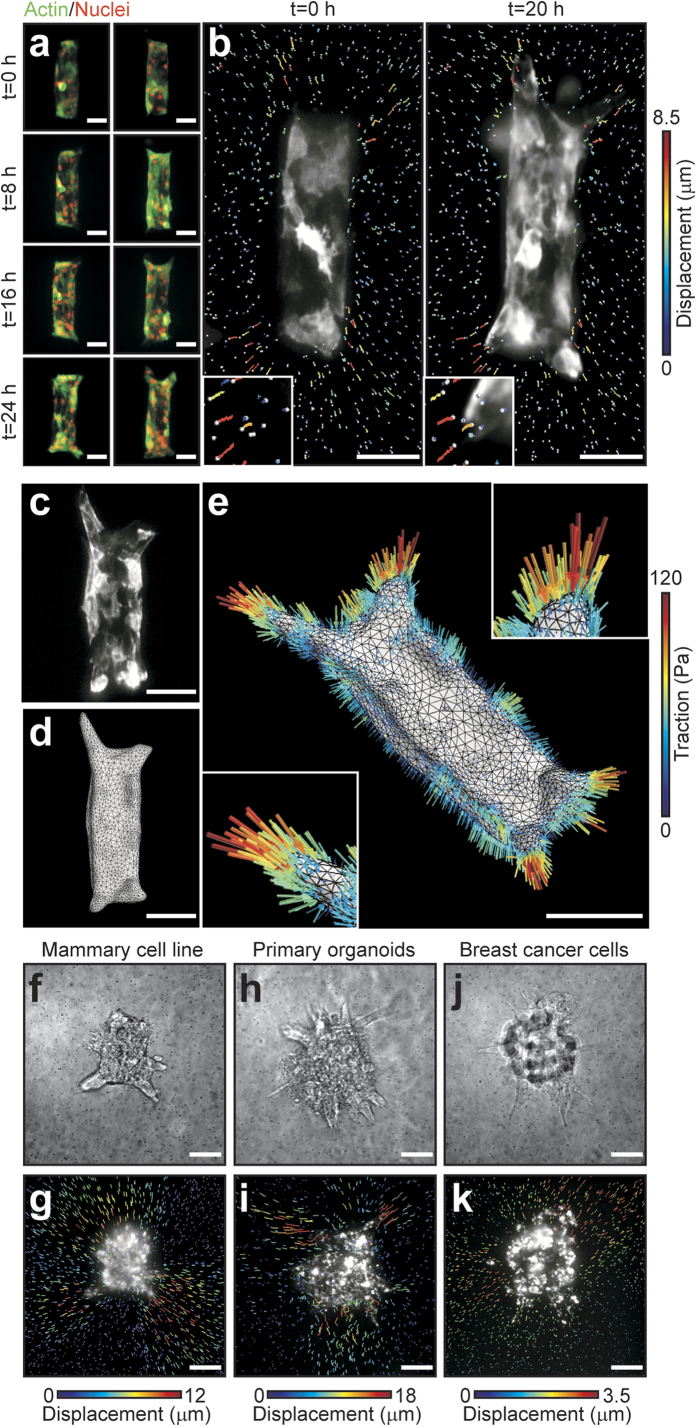
Epithelial cells migrate collectively by exerting tensile forces on the surrounding 3D matrix. (**a**) Confocal fluorescence images showing collective migration of mammary epithelial tissues labeled with LifeAct-GFP (green) and H2B-mCherry (red) in type I collagen gels over 24 hours. Images are representative of three independent replicates in which >50 tissues were monitored. (**b**) Confocal slice of tissues labeled with LifeAct-GFP at 0 and 20 hours. Resulting displacements of >100 beads embedded in the matrix are superimposed. Images are representative of four independent replicates in which >50 tissues were monitored. (**c**) Confocal stacks of a tissue labeled with LifeAct-GFP were used to reconstruct (**d**) the surface of the tissue and the migrating cohorts. (**e**) A map of estimated traction forces exerted by collectively migrating epithelial cells. The displacements of >1000 beads embedded within the matrix were used to estimate the traction forces exerted by the tissue during collective migration. Images and displacements are representative of three independent replicates. (**f**) Phase contrast image of a cluster of mammary epithelial cells in a type I collagen gel undergoing collective migration. Image is representative of three independent replicates in which >5 tissues were monitored. (**g**) Confocal slice of the collectively migrating mammary epithelial cell cluster in (**f**) labeled with LifeAct-GFP. Resulting displacements of >100 beads embedded in the matrix are superimposed on the image. (**h**) Phase contrast image of a primary mammary organoid in a type I collagen gel undergoing collective migration. Image is representative of three independent replicates in which >5 organoids were monitored. (**i**) Confocal slice of the collectively migrating primary mammary organoid in (**h**) labeled with LifeAct-GFP. Resulting displacements of >100 beads embedded in the matrix are superimposed on the image. (**j**) Phase contrast image of a cluster of breast cancer cells in a type I collagen gel undergoing collective invasion. Image is representative of three independent replicates in which >20 clusters were monitored. (**k**) Confocal slice of the cluster of invasive cancer cells in (**j**) labeled with LifeAct-GFP. Resulting displacements of >100 beads embedded in the matrix are superimposed on the image. Scale bars, 50 μm.

We used arrays of microfabricated tissues to investigate the physical mechanisms that drive invasive collective migration. This approach generates hundreds of regularly spaced 3D epithelial tissues of defined size and shape, embedded in a matrix of native type I collagen^18^. In this system, cells invade collectively from predictable and reproducible locations within the tissues (Fig. 1a), enabling high-throughput analysis with high statistical confidence^16^,^18^. Importantly, unlike classic *in vivo* models, these platforms enable the control, measurement, and manipulation of mechanical parameters.

To measure matrix deformations and the corresponding mechanical forces that accompany collective cell migration, we monitored the motion of fluorescent beads embedded within the collagen surrounding the tissues. As cells invaded collectively into the collagen, the beads were displaced incrementally toward the extending cohort (Fig. 1b; Supplemental Movie 1), suggesting that the invading cohort generated a tensile force, which pulled on the surrounding matrix. To characterize these forces, we reconstructed the surface of the tissue (Fig. 1c,d), quantified bead displacements, and estimated the associated strains and tractions (adhesive forces that arise at the interface between the cells and the ECM). It should be noted that the goal of the quantification was to assess the spatial distribution of the forces and their directionality, rather than to provide an absolute measure of magnitude. Absolute quantification in this case is challenging, owing to the spatial and temporal variations in matrix mechanics^19^,^20^, as well as cell-induced heterogeneities and anisotropies (i.e. collagen alignment, discussed below). Tractions were localized to the invasive front (Fig. 1e), and, importantly, were tensile in nature, indicating that the cohort translocated forward by pulling on the matrix.

To determine the generality of this physical mechanism, we measured matrix displacements in classic models of mammary epithelial branching morphogenesis, itself a form of collective migration^21^,^22^. Branch initiation and extension from clusters of mammary epithelial cells (Fig. 1f,g) and primary mammary organoids (Fig. 1h,i) were accompanied by inward-directed displacements, which again localized to narrow (~50 μm wide) regions ahead of each branch and propagated up to 150 μm away. Collective invasion of cancer cells also proceeded via a pulling mechanism (Fig. 1j,k). However, displacements in this case were considerably smaller and more diffuse than those arising during collective migration of non-neoplastic epithelial cells.

**Figure 2:**
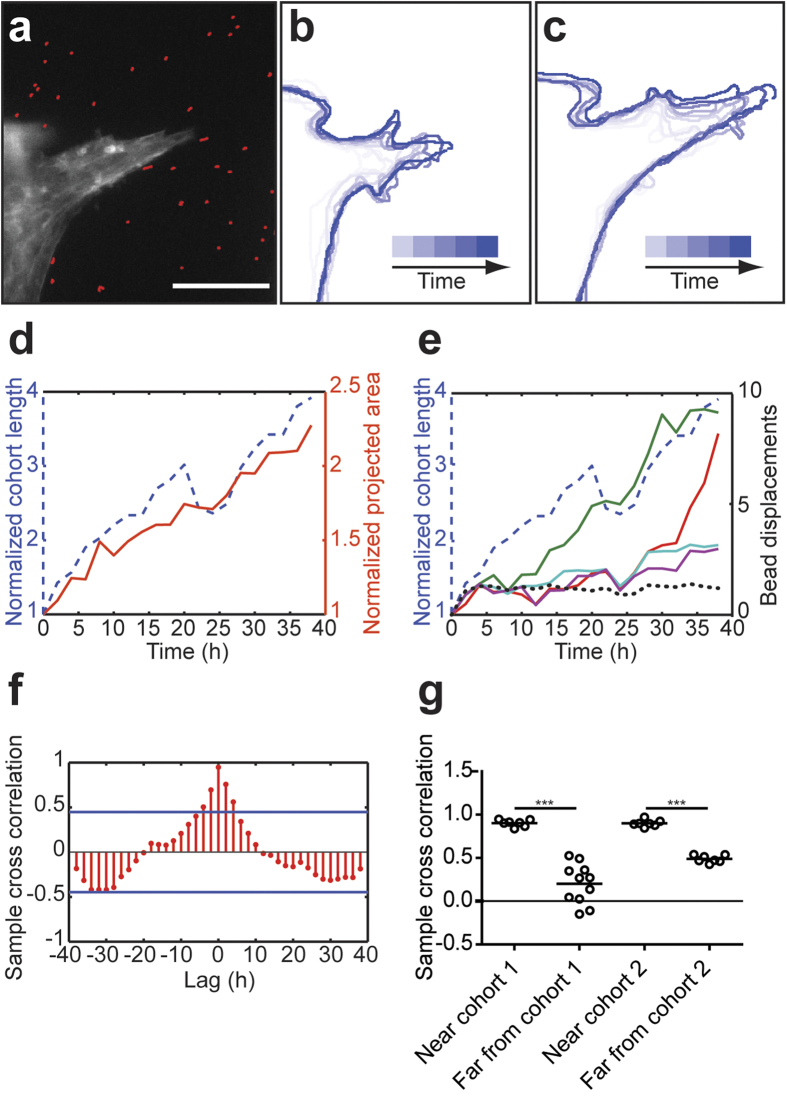
Collectively migrating cohorts exhibit dynamic changes in shape, which are in phase and correlated with surrounding matrix deformations. (**a**) Confocal fluorescence image of a collectively migrating cohort after 40 hours of live imaging with bead displacements superimposed (red). Image is representative of 4 independent replicates. Within a single experiment, four tissues were monitored. Contours of the migrating cohort in (**a**) from (**b**) 0 to 20 hours and from (**c**) 20 to 40 hours; darker colors represent later time points. (**d**) Plot of normalized cohort length (blue, dashed line) and projected area (red, solid line) for the cohort in (**a**). (**e**) Plot of normalized cohort length (blue, dashed line) and displacements (various colors, solid lines) of beads near (<50 μm) the migrating cohort in (**a**). Displacements are representative of 18 tracks analyzed. Also included is the displacement (black, dotted line) of one bead located far (>50 μm) from the migrating cohort. (**f**) Sample cross correlation plot comparing variations in cohort length to the displacement of a bead located near the migrating cohort (sample cross covariance). Plot is representative of 18 displacement tracks analyzed. (**g**) Cross correlation coefficients comparing temporal change in cohort length to the displacement trajectories of fluorescent beads near the cohort and far from the cohort for two separate representative samples. Mean of four replicates is shown. Beads near cohort 1: n = 7; beads far from cohort 1: n = 11; beads near cohort 2: n = 7; beads far from cohort 2: n = 7. ***P < 0.001, Student’s *t*-test. Scale bar, 50 μm.

Live imaging revealed the dynamics of collective migration and the interactions between the cells and their surrounding ECM (Fig. 2a). Collectively migrating cohorts exhibited dynamic changes in shape during migration (Fig. 2b,c). The projected area of the cohorts increased relatively linearly in time, while their lengths fluctuated (Fig. 2d). Comparing bead displacements to changes in cohort length revealed that beads adjacent to the extending collective moved coordinately and in phase with the cohort (Fig. 2e–g). Beads far from the migrating cohort (>50 μm) showed little displacement, and their movements did not correlate with that of the cohort (Fig. 2g). We also noted that cohorts continuously exert a tensile force on the ECM during extension, holding the ECM taut during migration (Supplemental Movie 2).

**Figure 3:**
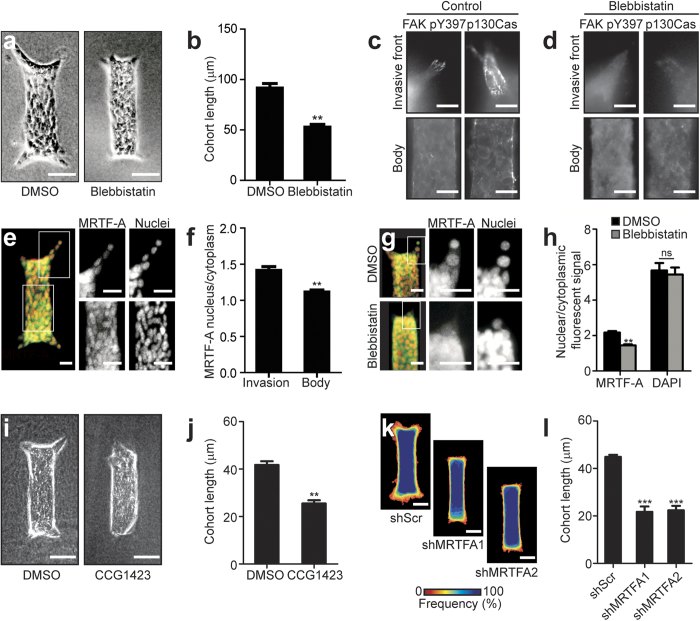
Tensile forces drive collective migration by activating mechanically sensitive intracellular signaling and transcription factors. (**a**) Phase contrast images showing collective migration from representative tissues treated with DMSO (control) or blebbistatin (12.5 μM). (**b**) Quantification of cohort length from tissues treated with DMSO or blebbistatin. Mean ± s.e.m. of three replicates is shown, n = 60 tissues per group. **P < 0.01, Student’s *t*-test. Immunofluorescence staining for FAK pY397 and phospho-p130Cas in representative DMSO (**c**) and blebbistatin-treated (**d**) migrating cohorts. (**e**) Confocal image showing immunofluorescence staining for MRTF-A localization (green) and nuclei (red) in a representative tissue. (**f**) Quantification of the localization of MRTF-A (nucleus/cytoplasm) in tissues. Levels of nuclear and cytoplasmic MRTF-A were quantified by measuring signal intensity in the two compartments. Mean ± s.e.m. of three replicates is shown. Migrating cohort (invasion): n = 14; quiescent body: n = 11. **P < 0.01, Student’s *t*-test. (**g**) Confocal image showing immunofluorescence staining for MRTF-A localization (green) and nuclei (red) in representative tissues treated with DMSO or blebbistatin. (**h**) Quantification of the localization of MRTF-A (nucleus/cytoplasm) in tissues treated with DMSO or blebbistatin. Levels of nuclear and cytoplasmic MRTF-A were quantified by measuring signal intensity in the two compartments. Mean ± s.e.m. of three replicates is shown. MRTF-A DMSO: n = 29; MRTF-A blebbistatin: n = 21; DAPI DMSO: n = 10; DAPI blebbistatin: n = 12. **P < 0.01, Student’s *t*-test. (**i**) Phase contrast images showing collective migration from representative tissues treated with DMSO or CCG-1423 (10 μM). (**j**) Quantification of cohort length from tissues treated with DMSO or CCG-1423. Mean ± sem. of three replicates is shown. Invading cohorts treated with DMSO: n=80; invading cohorts treated with CCG-1423: n = 57. **P < 0.01, Student’s *t*-test. (**k**) Frequency maps showing collective invasion from 34 tissues (three independent replicates) transfected with scrambled shRNA (shScr) or shMRTF-A (two constructs). (**l**) Quantification of cohort length from tissues transfected with shScr or shMRTF-A. Mean ± s.e.m. of three replicates is shown, n = 50 tissues per group. ***P < 0.001, Student’s *t*-test. All images are representative of three independent replicates in which >50 tissues were monitored. Scale bars, 50 μm (**a**,**i**,**k**), 25 μm (**c**,**d**,**e**,**g**).

To determine whether active cellular contractility was required for collective migration, we blocked cytoskeletal tension by treating the invading tissues with blebbistatin (Fig. 3a) or Y27632 (Fig. S1a), which inhibit myosin ATPase and Rho kinase, respectively. Disrupting cell-generated forces significantly impaired the extent of collective migration (Fig. 3b; Fig. S1b). Conversely, enhancing cellular contractility by treating with LPA, an activator of Rho, increased the extent of migration (Fig. S1a).

Mechanical forces generate cellular deformations and propel cell movements during development and disease progression^23^. In addition to their physical roles, mechanical forces have signaling and regulatory functions^24^. To examine whether the tensile forces arising from the leading edge of the invading cohorts serve a role beyond facilitating physical translocation, we visualized signaling through focal adhesion kinase (FAK) and p130Cas, both of which are activated downstream of integrins in response to mechanical force^25^,^26^,^27^. Immunofluorescence analysis revealed enhanced activation of both FAK and p130Cas within the extending cohorts; both localized to discrete matrix adhesions (Fig. 3c). Consistently, disrupting cytoskeletal tension with blebbistatin abolished activation of FAK and p130Cas within the cohorts (Fig. 3d).

Mechanical tension and signaling through FAK have also been shown to promote the nuclear translocation of myocardin-related transcription factor (MRTF)-A^28^, a co-factor for serum response factor (SRF). Moreover, the collective migration of border cells during *Drosophila* oogenesis is driven by tension-mediated activation of SRF and MRTF-A^29^. Tensile forces generated during collective migration induce nuclear translocation of MRTF-A, its association with SRF, and subsequent transcription of SRF-target genes, regulating differentiation, proliferation, and motility^29^. MRTF-A is also required for cancer cell migration^30^,^31^ and branching morphogenesis of the *Drosophila* trachea^32^, and is regulated by tension in mammalian epithelial cells^33^,^34^. To test whether invasive collective migration of mammalian tissues is regulated by MRTF-A in a tension-dependent manner, we used immunofluorescence to characterize its localization (Fig. 3e). MRTF-A was mainly nuclear in cells within the invading cohorts, whereas in quiescent tissue it was both nuclear and cytoplasmic (Fig. 3f). To determine whether the nuclear translocation of MRTF-A was force-dependent, we altered tension in the cells by treating with blebbistatin (Fig. 3g) or by controlling their distention using micropatterning (Fig. S2a). Pharmacologically abolishing cytoskeletal tension significantly attenuated the nuclear translocation of MRTF-A (Fig. 3h), as did reducing tension by restricting cell spreading (Fig. S2a,b). Treating the tissues with CCG1423, which blocks the nuclear translocation of MRTF-A^35^, significantly impaired invasion (Fig. 3i,j), as did shRNA-mediated depletion of MRTF-A (Fig. 3k,l, S2c). These data suggest that increased tension within the invading cohort causes nuclear translocation of MRTF-A to promote collective migration.

**Figure 4:**
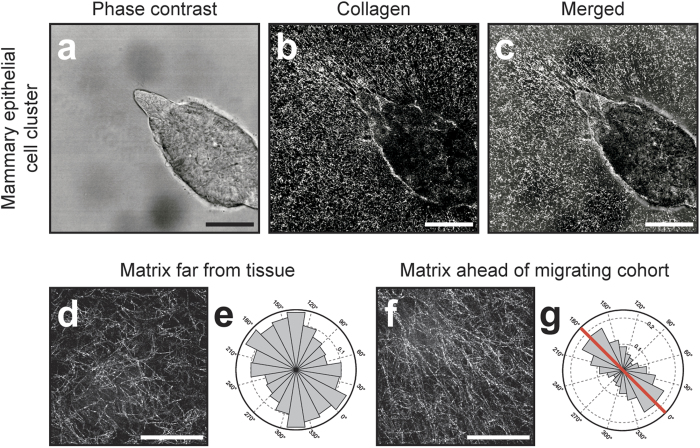
Tensile forces remodel matrix at the leading edge of migrating cohorts. (**a**) Representative phase contrast image showing cells invading from a mammary epithelial cell cluster. (**b**) Confocal reflection image depicting the structure of the collagen matrix surrounding the tissue in (**a**). (**c**) Merged image of (**a**) and (**b**). High magnification images showing the structure of the collagen (**d**) far from the invading tissue in (**a**) and (**f**) adjacent to the invasive front of the tissue in (**a**). (**e**) Rose plot showing fibril angles far from the tissue in (**a**). Histogram represents fiber angles computed at 900 image locations within (**d**). (**g**) Rose plot showing fibril angles adjacent to the invasive front of the tissue in (**a**). Histogram represents fiber angles computed at 900 image locations within (**f**). Red line indicates the direction of collective invasion. All images are representative of three independent replicates. Scale bars, 50 μm (**a**–**c**), 25 μm (**d**,**f**).

It has been proposed that during collective invasion through collagenous matrices, cells follow paths of least resistance created by proteolytic degradation and softening of the ECM^36^. To test for such a mechanism, we used confocal reflection microscopy (CRM) to visualize the structure of the matrix surrounding the invading cohorts (Fig. S3a). We observed no obvious proteolytic remodeling ahead of the leading edge (Fig. 4a-c; Fig. S3a–c). Instead, CRM revealed a different kind of matrix remodeling at these locations: collagen fibrils were compacted and aligned into parallel and highly directional tracks emanating from the invasive front and propagating over distances spanning ~100 μm from the tissue (Fig. 4c). Measuring the angles of collagen fibrils revealed that those far from the tissue (Fig. 4d) were distributed randomly (Fig. 4e), while those ahead of the migrating cohort (Fig. 4f) oriented preferentially in the direction of migration (Fig. 4g). Imaging the matrix around primary organoids similarly revealed that collagen was compacted into dense and directionally oriented fibrils from the leading edge of extending branches (Fig. S3e–g). Blocking cytoskeletal tension prevented collagen alignment ahead of the migrating cohorts (Fig. S3i), suggesting that alignment was mediated by migration-generated tensile forces.

**Figure 5:**
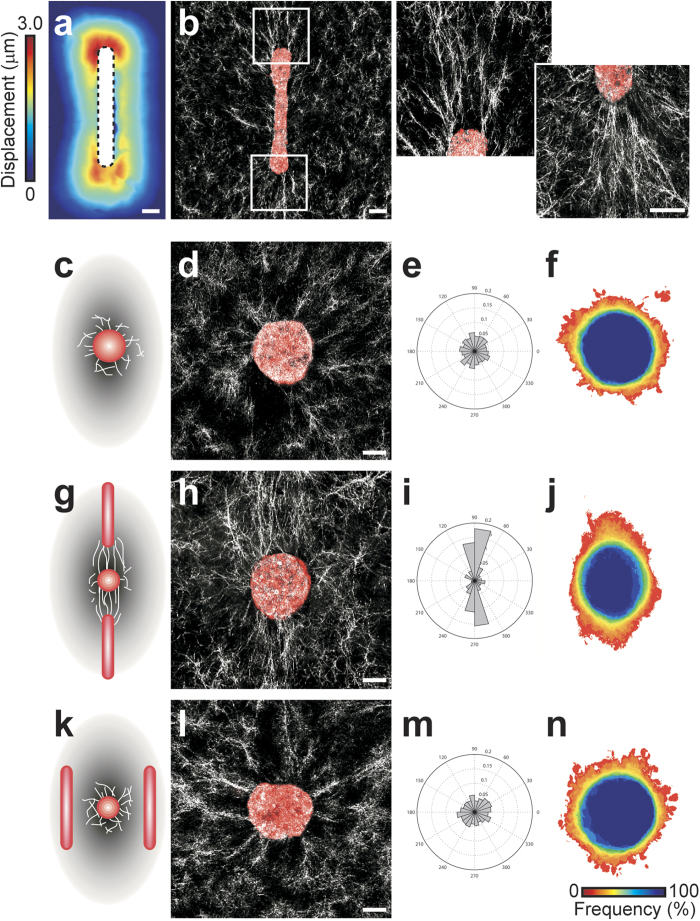
Matrix alignment spatially directs collective migration. (**a**) Heat map showing matrix deformation around rectangular tissues. The average magnitude of matrix deformation arising from forces generated by >10 microfabricated tissues is shown. Matrix deformations caused by a single tissue were determined by tracking >100 beads embedded in the surrounding collagen. Heat map is representative of >20 independent replicates. (**b**) Confocal reflection image showing the structure of collagen surrounding a representative rectangular epithelial tissue labeled with DiI (red) before the tissue undergoes collective migration. Also shown are high magnification images of regions of fibril alignment near the tips of the rectangular tissue. (**c**) Schematic and (**d**) confocal reflection image of collagen surrounding a representative circular tissue labeled with DiI (red) before the tissue undergoes collective migration. (**e**) Rose plot showing the angles of collective invasion from 66 circular tissues (three independent replicates). (**f**) Frequency map representing collective migration from 66 circular tissues (three independent replicates). (**g**) Schematic and (**h**) confocal reflection image of collagen surrounding a representative circular tissue labeled with DiI (red) exposed to regions of fibril alignment before the tissue undergoes collective migration. (**i**) Rose plot showing the angles of collective invasion from 83 tissues (three independent replicates) in the configuration shown in (**g**). (**j**) Frequency map representing collective migration from 83 tissues (three independent replicates) in the configuration shown in (**g**). (**k**) Schematic and (**l**) confocal reflection image of collagen matrix surrounding a representative circular tissue labeled with DiI (red) proximal to rectangular tissues, but not exposed to regions of preferential fibril alignment before the tissue undergoes collective migration. (**m**) Rose plot showing the angles of collective invasion from 53 tissues (three independent replicates) in the configuration shown in (**k**). (**n**) Frequency map representing collective migration from 53 tissues (three independent replicates) in the configuration shown in (**k**). All images are representative of three independent replicates. Scale bars, 50 μm.

The generation of physiologically functional tissue geometries during epithelial morphogenesis requires tight spatial guidance of collective cell movements. Hence, it is necessary to determine the guidance cues that initiate and propel movement, as well as those that confer and maintain directionality. Classically, guidance roles have been attributed to soluble cues, including growth factors and various chemokines^37^,^38^,^39^. Recently, however, long-range transmission of mechanical signals has been proposed to independently guide collective cell migration in the context of epithelial tubulogenesis^40^,^41^. Cells migrate more efficiently through directionally aligned fibrillar matrices than randomly oriented ones^42^,^43^. Therefore, we postulated that tension-mediated alignment ahead of the invasive front facilitates further collective migration and provides directionality to the movement. To test these hypotheses, we incorporated epithelial tissues into regions of pre-aligned ECM (of length scales similar to those ahead of the leading edge of migrating cohorts). Mechanical strains generated by tissues of non-circular geometries are non-uniformly distributed within the surrounding matrix^16^,^44^ (Fig. 5a). CRM revealed that the ECM was preferentially remodeled and aligned in regions experiencing high strains (Fig. 5b). In contrast, tissues of circular geometry experienced no spatial variations in the structure or alignment of the surrounding ECM (Fig. 5c,d). Consistently, collective invasion from these circular tissues occurred without directional preference (Fig. 5e,f). However, when rectangular and circular tissues were juxtaposed to align the ECM at specific locations around the latter (Fig. 5g,h), a directional bias emerged: cohorts from the circular tissues migrated preferentially in the direction of aligned fibrils (Fig. 5i). Furthermore, cohorts migrating along aligned fibrils were longer and contained more cells than did those migrating through randomly oriented matrix (Fig. 5j). To rule out chemoattraction as a possible explanation for the migration bias, we altered the relative configuration^18^ such that rectangular tissues no longer aligned the ECM surrounding the circular tissues (Fig. 5k,l). The migration bias disappeared (Fig. 5m,n), confirming that the directional cue was provided by ECM alignment and not soluble factors. These data indicate that matrix alignment plays two roles during collective migration: it increases the efficiency of migration and spatially directs migrating cohorts.

## Discussion

Together, these results reveal an essential and multifaceted role for endogenous mechanical forces during collective migration through 3D fibrillar matrices. We characterized and made quantitative estimates of the tensile forces that arise during this process, the nature and existence of which had only been inferred thus far^5^. We showed that migrating collectives propel themselves through fibrillar matrices by pulling on impeding fibers. Furthermore, our data suggest that this physical mode is a general migration strategy, as functionally normal epithelial cells, cancer cells, and primary organoids moved collectively using a similar mechanism. Ours is the first study to examine the temporal and spatial dynamics of physical forces exerted by a migrating cohort fully embedded in 3D matrix. Importantly, our data show that the cohort does not exert continuous tensile force on the surrounding ECM. Instead, the multicellular collective frequently releases its grip on the matrix or relaxes its hold as it extends (Supplemental Movie 2). As a consequence, the length of the migrating cohort does not increase monotonically; rather, periods of extension are followed by periods of retraction. Subcellular pulsatile tensile force has been observed in filopodial extensions of single migrating cells^45^, but cellular-level pulsatility has not been reported previously in studies of the 2D collective migration of monolayers of cells^13^,^46^, and may be a specific feature of collective migration in 3D. Indeed, protrusion and retraction were recently reported in the collective migration of *Drosophila* border cells^47^, although mechanical forces per se were not measured in that study.

Importantly, the forces that arise during collective migration have both physical and signaling roles. In addition to providing ‘grip’ and ‘pull’ to enable translocation, these tensile forces regulate the efficiency and direction of migration by conditioning both intracellular components and the ECM. The mechanosensitive focal adhesion proteins FAK and p130Cas, both regulators of cytoskeletal tension, motility, and invasiveness, were preferentially activated in a force-dependent manner within the invasive front of the migrating cohorts. These results are consistent with studies in 2D epithelial monolayers in which cytoskeletal tension in regions of high curvature was found to be important for leader cell formation during collective migration^46^. We also found that mechanical force regulated nuclear localization, and likely transcriptional activation of MRTF-A, which was required for collective migration. Notably, MRTF-A regulates genes that encode cytoskeletal and adhesion proteins involved in the force-generating machinery of the cell^30^. Accordingly, it is possible that intracellular forces and MRTF-A engage in a dialogue of positive feedback, ultimately increasing the efficiency of collective migration. It will be interesting to determine how the temporal changes in force discussed above correlate with the dynamics of molecular signaling, both at cell-matrix and cell-cell junctions, as well as downstream of Rho and Rac small GTPases; a recent study reported that E-cadherin adhesions between border cells and nurse cells in the *Drosophila* ovary participate in a positive feedback loop with Rac and actin assembly to stabilize directed collective migration^47^.

Mechanical forces facilitate collective migration not only by influencing the cells themselves, but also by priming the surrounding ECM. We found that the matrix preceding a migrating cohort is remodeled into parallel fibrils in a process requiring mechanical force. Proteolysis may be required as a means to accommodate the growing structure^48^ rather than to create paths along which migration occurs, though pericellular proteolysis may assist in ECM remodeling. Others have shown that collagen fibrils can be aligned via mechanical strain even in the absence of cells^49^. Here, directionally aligned collagen fibrils increase the efficiency of collective migration by providing a path for persistent motility. Our findings are consistent with models suggesting that collective migration follows paths of least resistance^36^. However, our data show that these paths are generated primarily via physical forces, and that tissue geometry plays a role in the localization of these forces and subsequent ECM alignment. Moreover, these results are consistent with recent studies demonstrating that mammary acini can mechanically align and concentrate surrounding ECM fibrils over long distances^50^. Consistently, contractility-mediated local collagen reorganization at the tumor-stroma interface has been shown to promote cancer cell invasion^10^,^42^,^43^,^51^. A recent study demonstrated enhanced collagen alignment proximal to the terminal end buds of the mouse mammary gland, and suggested that these patterned collagen fibers orient the branching mammary epithelium^52^. Despite evidence showing that Rho-mediated contractions are required for collagen remodeling^43^,^52^, it is unclear how matrix alignment is restricted to the leading edge of a globally contracting tissue. We found that the tensile forces driving migration are highly localized (Fig. 1e,g,i,k) and responsible for generating the restricted patterns of matrix alignment. The spatial distribution of forces is in turn controlled by the geometry of the cohort: by engineering tissues of tubular geometry, we were able to restrict both contractile forces and collagen alignment to a specific region of the tissue (Fig. 5a,b). The resulting narrow (50–100 μm) strips of aligned collagen successfully controlled the directionality of migrating cohorts. An important implication of these findings is that the geometries of tissues generated through collective migration may be self-referential: the initial geometry dictates the emergent geometry by mechanically controlling the pattern of matrix remodeling.

Notably, although normal epithelial cells and invasive cancer cells migrated collectively via similar physical mechanisms, we observed striking differences in the spatial organization of the forces and resulting matrix remodeling (Fig. 1f,k). In particular, whereas collective migration of non-neoplastic cells generated highly restricted and directional force fields and matrix alignment, the forces produced by migrating cancer cohorts appeared to be diffuse and delocalized. Comprehensive understanding of the differences between collective cell movement during morphogenesis and cancer progression will require dynamic spatiotemporal mapping of the force fields and matrix remodeling associated with the two types of processes *in vivo*.

## Methods

### Cell culture and reagents

Functionally normal EpH4 mouse mammary epithelial cells (ATCC) were cultured in 1:1 DMEM:F12 supplemented with 2% fetal bovine serum (FBS; Atlanta Biologicals), 5 μg/ml insulin, and 50 μg/ml gentamicin (Sigma). MDA-MB-231 human breast cancer cells (ATCC) were cultured in 1:1 DMEM:F12 supplemented with 10% FBS and 50 μg/ml gentamicin. Primary mammary epithelial organoids were prepared from 8-week-old CD1 mice (Charles River) as described previously^53^. Mice were housed and euthanized in accordance with protocols approved by the Institutional Animal Care and Use Committee at Princeton University. Isolated organoids were resuspended in 1:1 DMEM:F12 supplemented with 10% FBS, 5 ng/ml EGF, insulin/transferrin/sodium selenite (ITS; Sigma), and penicillin/streptomycin (Sigma), and embedded immediately in collagen gels. Cells were maintained in a 37 °C incubator with 5% CO_2_. The following reagents were used at the concentrations indicated: blebbistatin (12.5 μM; Sigma), CCG1423 (10 μM; Cayman Chemicals), Y27632 (20 μM; Tocris), lysophosphatidic acid (LPA, 10 μg/ml; Cayman Chemicals) and were added to the medium 1−4 hours after tissues began migrating collectively.

### Microfabricated tissues and 3D culture models

3D epithelial tissues were constructed as described previously^54^. Briefly, neutralized non-pepsinized native type I collagen (6 mg/ml; BD Biosciences) was gelled at 37 °C against a stamp of poly(dimethylsiloxane) (PDMS; Sylgard 184, Ellsworth Adhesives) to generate micrometer-scale cavities of defined geometry. Mammary epithelial cells were allowed to settle within the cavities and a second layer of collagen was placed on top of the gel. Medium supplemented with HGF (10 ng/ml) was added to the samples ~20 hours later; collective migration began 24−48 hours after the addition of HGF. Matrix deformations were visualized by incorporating 1-μm diameter fluorescent polystyrene beads (Invitrogen) in the collagen solution (~4 × 10^8^  beads/ml). Clusters of mammary epithelial or tumor cells were prepared by shaking overnight (170 rpm at 37 °C for 14 hours) in the presence of 0.083% (w/v) pluronic F108 (BASF). Clusters of cells ~100 μm in diameter were collected by brief centrifugation (200 rpm for 1 min) and embedded within 6 mg/ml of type I collagen as described previously^53^. A cell-free layer of collagen was included beneath the layer containing clusters. Medium supplemented with HGF (10 ng/ml) was added to the samples.

### Immunofluorescence analysis

Samples were washed in PBS and fixed in 4% paraformaldehyde for 15 min at room temperature. Samples were permeabilized twice for 10 min in 0.5% Igepal Ca-630 and incubated in 0.1% Triton X-100 in PBS for 15 min. Samples were blocked overnight at 4 °C in 10% goat serum in PBS (PBS-S), followed by overnight incubation at 4°C in primary antibody against FAK pY397 (Invitrogen 44–624G), phospho-p130Cas (Cell Signaling 4015S), or MRTF-A (Sigma HPA030782) at 1:100 dilution in PBS-S. Samples were washed with PBS and incubated in secondary antibody at 1:1000 in PBS-S overnight at 4 °C.

### Constructs, transfection, and transduction

Mouse pLKO.1 lentiviral MKL1 shRNA (shMRTF-A#1) and shMRTF-A#2 were obtained from Open Biosystems. Mouse pLKO.1 lentiviral scrambled shRNA was obtained from AddGene. Cells were transfected using Fugene HD (Roche). To visualize collective movements, epithelial cells were transduced with a recombinant adenovirus encoding LifeAct-GFP^55^ and/or H2B-mCherry (Vector Biolabs) at an MOI resulting in >99% transduction efficiency.

### Quantitative image analysis

The length of collectively migrating cohorts was measured from the edge of the original tissue to the tip of the migrating cohort using ImageJ software (NIH). The levels of nuclear and cytoplasmic MRTF-A in confocal slices of stained samples were quantified by measuring the signal intensity in the two compartments using ImageJ. Frequency maps of collective migration were created using binarized images of ~50 tissues of identical initial geometry. These were stacked using Scion Image software to obtain a pixel frequency map and color-coded in Adobe Photoshop.

### Real-time microscopy and measurement of matrix displacements

Time-lapse movies were collected using a Hamamatsu ECCD camera attached to a Nikon Ti-U inverted microscope customized with a spinning disk (BD Biosciences) and fitted with a humidified environmental chamber held at 37 °C and 5% CO_2_. Fluorescent beads and LifeAct-GFP-labeled cells were imaged simultaneously. Confocal stacks (390 × 390 × 100 μm, spaced 1 μm in the z-direction) were acquired using a Plan Apo 20× 0.4 *NA* objective every 2 hours beginning at 24 hours after initial microfabrication and addition of HGF for a total of 20–48 hours. To measure matrix deformations at a given time during the migration process, bead positions were recorded before and after lysing the collectively migrating tissue using 0.1% (w/v) of Triton X-100. 3D and bead displacements were extracted using the Autoregressive Motion tracking algorithm in the image analysis software Imaris^®^ (Bitplane). The displacement gradient matrix for finite strains was used to calculate tissue-induced strains within the collagen gel:


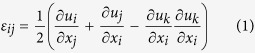


### Mechanical properties and constitutive model of collagen gels

Material properties of the collagen gels were determined via bulk rheometry using the cone-and-plate setup on a Physica MCR 501 rheometer (Anton Paar). The chamber was held at 37 °C and 100% humidity using a Peltier plate and humidity chamber to mimic experimental conditions and to prevent the collagen from drying. To rigorously compute the mechanical stresses exerted by the migrating cohort, the anisotropic, viscoelastic behavior of the collagen matrix must be considered. In this study, however, we were not concerned with the absolute magnitude of the computed stresses; rather, we sought to estimate the spatial and temporal distributions of mechanical stress with respect to the migrating cohort, and (as a first approximation) made several simplifying assumptions. Hence, Hooke’s law for isotropic materials was used to describe the constitutive behavior of the collagen gels during tissue-induced deformation:


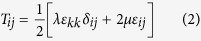



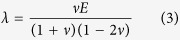



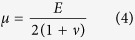


where δ_*ij*_ is the Kronecker delta, *T* is the Cauchy stress tensor, μ and *λ* are the Lamé parameters, *E* is Young’s modulus and *ν* =0.2 is the Poisson ratio^56^.

### Epithelial tissue surface reconstruction and mesh generation

The surface of the branched epithelium at a snapshot in time (24 hrs after branch induction) was reconstructed from 3D confocal stacks of LifeAct-GFP-transduced cells. Image segmentation was performed manually in ImageJ to define the cellular portion of the 3D stack. A 3D surface was subsequently generated using Amira^®^ (Visage Imaging) and converted to a parasolid object using Mesh2Solid (Sycode). The solid was imported into Comsol Multiphysics 4.2a (Comsol Inc.) and enclosed within a second computational domain of cylindrical geometry (2 mm in height and diameter) representing the collagen gel. A quadratic tetrahedral finite element mesh of the epithelial surface and the surrounding gel was generated.

### Calculation and reporting of mechanical stress

The equations of motion, the displacement gradient matrix (Equation 1), and Hooke’s law for isotropic materials (Equations 2), were used to calculate the Cauchy stress tensor throughout the domain, as described previously^16^. The boundary conditions were as follows: displacements at the epithelium-matrix interface were interpolated from experimentally measured bead displacement values. A displacement of zero was assumed at the outer boundaries of the collagen gel. The components and the magnitude of the traction vector at a point on the epithelial surface were calculated as:


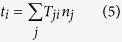



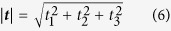


where *t*_*i*_, *i* = 1,2,3, are the components of the stress vector; *n*_*j*_, *j* = 1,2,3, are the components of the unit normal vector at a point on the epithelial surface; *T*_*ij*_ are the components of the Cauchy stress tensor, and 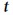 is the magnitude of the traction vector.

### Correlation analysis

Correlations between bead displacements and variations in the length of a migrating cohort were determined using the sample cross covariance function, which indicates the covariance between discrete sets of data collected over time, and defines whether there is a lag between the two time series. For two time series 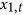 and 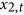, the lag 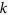 cross-covariance is estimated as:


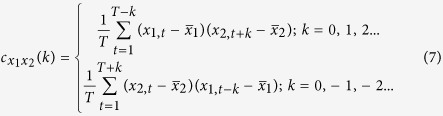


Here, 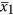 and 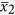 are the sample means of the time series. The cross correlation coefficient is:


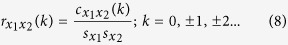


Where 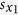 and 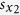 are the sample standard deviations of the time series. Lag 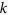 cross-covariances and cross correlation coefficients were calculated between the length variations of a migrating cohort and the displacements of beads within the surrounding ECM that were both adjacent to (<50 μm) and far from (>50 μm) the extending cohort.

### Confocal reflection microscopy

The fibrillar structure of the collagen was visualized using a Leica SP5 laser-scanning confocal microscope as described previously^16^. Epithelial tissues were fabricated as described above; cells labeled with DiI were embedded in a matrix composed of type I collagen and Matrigel (BD Biosciences) in a 1:4 ratio. Collagen matrices were illuminated with an Argon laser (488 nm) and imaged in reflection mode using a 20× or 63× oil-immersion objective. Images of the collagen surrounding tissues (prior to migration) were taken at a z-position corresponding to the middle of the tissue.

### Quantifying collagen fibril alignment

Collagen fibril alignment was quantified by measuring the orientation of pixel intensity gradients in subregions of the confocal reflection images. The results were displayed in a circular histogram^57^. To compare these fibril orientations (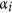) with branch angles (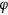) in cultured mammary organoids, we calculated the angle difference 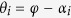 in the neighborhood of extending branches, where 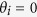 indicates fibers precisely aligned with an extending branch. As a control, fibril orientations (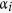) were also computed for regions of the collagen gel away from cultured organoids.

### Statistical analysis

Results were analyzed in GraphPad Prism (GraphPad Software). The two-tailed Student’s *t*-test and two-tailed Kruskal-Wallis test with Dunn’s multiple comparison post-test were used where appropriate. The alpha level was set at 0.05 for all statistical tests. The normality of data was confirmed prior to use of parametric tests.

## Additional Information

**How to cite this article**: Gjorevski, N. *et al.* Dynamic tensile forces drive collective cell migration through three-dimensional extracellular matrices. *Sci. Rep.* **5**, 11458; doi: 10.1038/srep11458 (2015).

## Supplementary Material

Supplementary Information

Supplemental Movie 1

Supplemental Movie 2
