# Supplementary material for: Dynamic tensile forces drive collective cell migration through three-dimensional extracellular matrices
Source: Sci Rep. 2015 Jul 13;5:11458. doi: 10.1038/srep11458 (PMC4499882; doi:10.1038/srep11458)
Supplement: Supplementary Information [file srep11458-s1.pdf]

Dynamic tensile forces drive collective cell migration through three-dimensional extracellular matrices

Nikolce Gjorevski[1†], Alexandra Piotrowski[1†], Victor D. Varner[1], and Celeste M. Nelson[1,2]*

Departments of [1]Chemical & Biological Engineering and [2]Molecular Biology

Princeton University, Princeton, NJ 08544

*Address correspondence to C.M.N.
303 Hoyt Laboratory, William Street
Princeton University
Princeton, NJ 08544
Tel: 609 258 8851
Fax: 609 258 1247
E-mail: celesten@princeton.edu

[†] These authors contributed equally to this work.

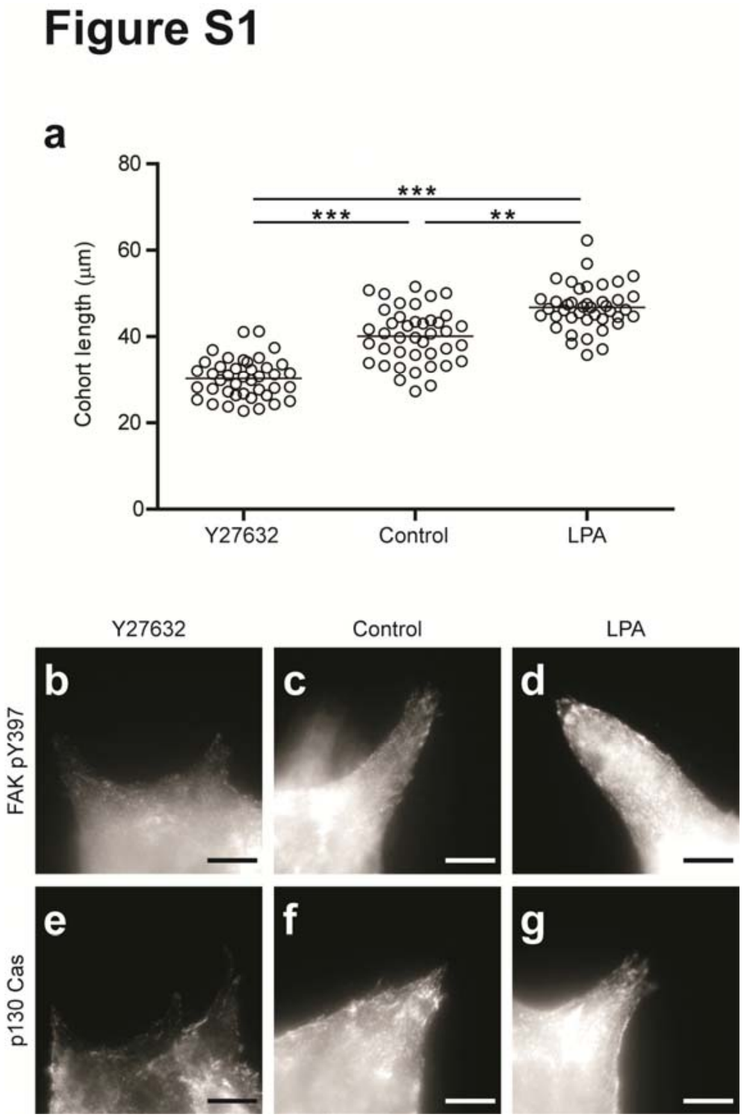

Figure S1: Tensile forces are required for collective migration and modulate activity of mechanosensitive proteins within the leading edge. (**a**) Quantification of cohort length from tissues treated with DMSO (control), Y27632 (20 μM), or LPA (10 μg/mL). Mean of three replicates is shown, n = 41 tissues per group. Significance level (Kruskal-Wallis test with Dunn's multiple comparison post-test) $^{**}P<0.01$, $^{***}P<0.001$. Immunofluorescence staining for FAK pY397 in representative (**b**) Y27632-treated, (**c**) control, and (**d**) LPA-treated migrating cohorts. Immunofluorescence staining for phospho-p130Cas in representative (**e**) Y27632-treated, (**f**)

control, and (**g**) LPA-treated migrating cohorts. All images are representative of three independent replicates. Scale bars, 25 μm.

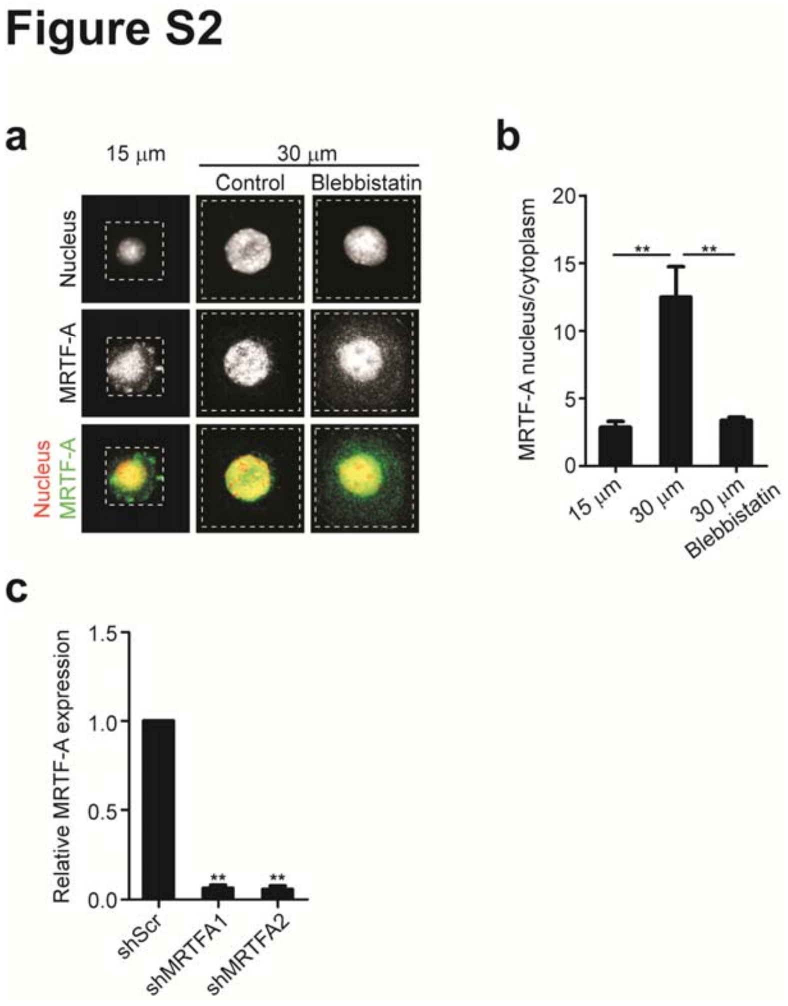

Figure S2: Nuclear translocation of MRTF-A is controlled by the tensional state of the cell. (**a**) Immunofluorescence staining for MRTF-A (greed) and nuclei (red) in representative epithelial cells plated on adhesive islands of varying area (15 μm or 30 μm squares) to control the extent of cell spreading. Tension within the highly spread cells was dissipated by treating with blebbistatin (25 μM). (**b**) Quantification of the localization of MRTF-A (nucleus/cytoplasm) in cells on 15 μm or 30 μm squares treated with DMSO (control) and cells on 30 μm squares treated with blebbistatin. The levels of nuclear and cytoplasmic MRTF-A were quantified by measuring the signal intensity in the two compartments. Mean ± s.e.m. of three replicates is shown, n=5 cells per condition. Significance level (Student's *t*-test) $^{**}P<0.01$. (**c**)

Transcript levels of MRTF-A in epithelial cells transfected with scrambled control shRNA (shScr) and shRNA against MRTF-A (shMRTFA, two constructs). Mean ± s.e.m. of three replicates is shown. Significance level (Student's *t*-test) $^{**}P<0.01$. All images are representative of three independent replicates.

Figure S3

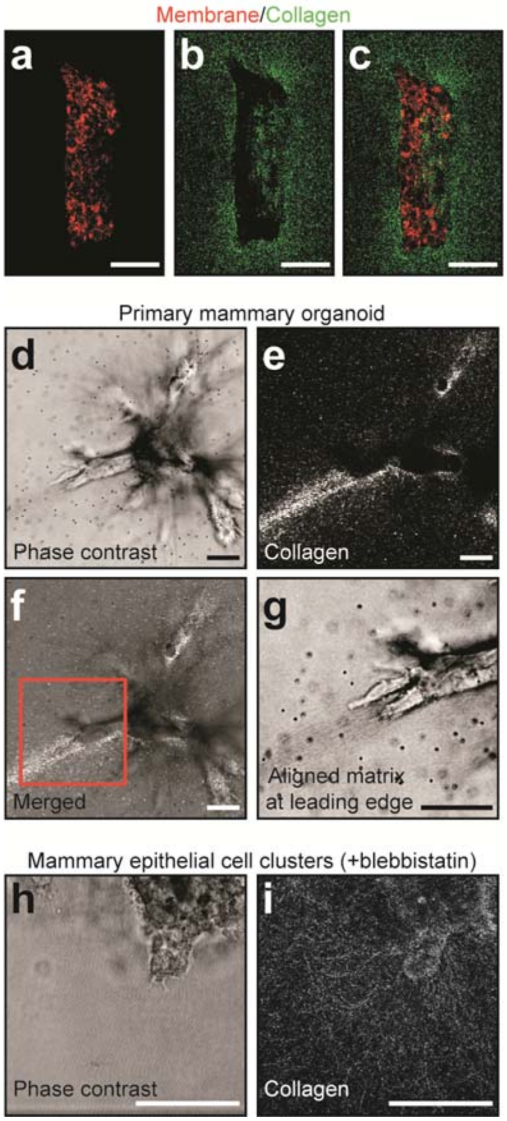

Figure S3: Tensile forces remodel collagen at the leading edge. (**a**) Confocal fluorescence image showing a representative collectively migrating epithelial tissue labeled with DiI (red). (**b**) Confocal reflection image showing collagen structure (green) around migrating cohort in (**a**). (**c**) Merged image of (**a**) and (**b**). (**d**) Phase contrast images of a representative mammary organoid. (**e**) Confocal reflectance image showing the structure of collagen surrounding the primary

mammary organoid in (**d**). (**f**) Merged image of (**d**) and (**e**). (**g**) High magnification image of a region in (**f**) showing aligned matrix at the leading edge of a migrating cohort. (**h**) Phase contrast image of a representative cluster of collectively migrating epithelial cells after treatment with blebbistatin. (**i**) Confocal reflection image showing the structure of collagen surrounding the cluster of collectively migrating mammary epithelial cells in (**h**). All images are representative of three independent replicates. Scale bars, 50 μm.

Supplemental Movie 1. Movie of multiple collective migration events from a single engineered tissue in which cells are labeled with LifeAct-GFP (green), and the resulting motion of the fluorescent beads (white) in the surrounding matrix, over a 24 hour time period. Movie is representative of three independent replicates.

Supplemental Movie 2. High magnification movie of a single collectively migrating cohort in which cells are labeled with LifeAct-GFP (green) and the resulting fluorescent bead motion (red) taken over a 40 hour time period. Note: this cohort is from a different tissue than that depicted in Supplemental Movie 1. Movie is representative of four independent replicates. Scale bar, 50 μm.
